# Identification of fungi in Tunisian olive orchards: characterization and biological control potential

**DOI:** 10.1186/s12866-020-01997-z

**Published:** 2020-10-12

**Authors:** Houda Gharsallah, Ines Ksentini, Sourour Naayma, Karama Hadj Taieb, Nour Abdelhedi, Christina Schuster, Mohamed Ali Triki, Mohieddine Ksantini, Andreas Leclerque

**Affiliations:** 1 Laboratory of Improvement and Protection of Genetic Resources of Olive Tree, Tunisian Olive Institute, University of Sfax, Airport Road, P.B. 1087, 3000 Sfax, Tunisia; 2 University of Sousse, Higher Institute of Agronomic Sciences of Chott Meriem, 4042 Sousse, Tunisia; 3 Institute for Microbiology and Biochemistry, Geisenheim University, Geisenheim, Germany; 4 Department of Biology, Technische Universität Darmstadt, Darmstadt, Germany

**Keywords:** Biological control, Fungi, Insect pests, Antagonism, Phytopathogenicity, Entomopathogenicity

## Abstract

**Background:**

Olive production is the main agricultural activity in Tunisia. The diversity of fungi was explored in two different olive groves located in two distant geographical zones in Sfax (Tunisia) with different management practices.

**Results:**

Fungal isolation was made from soil and the major olive tree pests, namely the Olive fly, *Bactrocera oleae* Gmelin (Diptera: Tephritidae), and the Olive psyllid, *Euphyllura olivina* Costa (Homoptera: Psyllidae). A total of 34 fungal isolates were identified according to their phenotypic, genotypic, biochemical and biological activities. Twenty fungal species were identified belonging to six different genera (*Alternaria*, *Aspergillus*, *Cladosporium*, *Fusarium*, *Lecanicillium* and *Penicillium*) by the analysis of their ITS1–5.8S–ITS2 ribosomal DNA region. Different bioassays performed in this work revealed that 25/34 (73.5%) of the identified fungal isolates showed an entomopathogenic and/or antagonistic activity, 9/34 (26.5%) of them displayed phytopathogenic features.

**Conclusions:**

Fungal species that showed entomopathogenic and/or antagonistic potentialities and that are non-phytopathogenic, (17/34; 50%) of our fungal isolates, could be explored for olive protection against fungal diseases and pests, and might have a future application as biocontrol agents.

## Background

Biological control is an often effective and environmentally friendly method for controlling pests and phytopathogens by a natural enemy and/or formulation. Some of these biological control methods as, e.g., combating insect pest species by pathogens and plant diseases by antagonists, were found efficient [1–3]. However, effectiveness is strongly related to the biological control agent’s origin, with native ones being more efficient and often achieve better results [4, 5]. Thus, it is important to ensure that the screening for insect pest pathogens and phytopathogenic antagonists is done in their original spreading areas.

The olive tree, *Olea europaea* L. (**Equisetopsida, Lamiales),** cultivation is among the important crops in the world [6]. Widespread areas are occupied by olive groves in the Mediterranean basin countries including Tunisia which ranks in the fourth position in terms of virgin olive oil production [6, 7]. In Tunisia, with approximately 8.5 million olive trees spreading over almost 1.7 million ha, Sfax governorate has the largest surface dedicated to olive cultivation, representing 18.7% of the national olive cultivation area [8], and ensuring 33% of the Tunisian olive oil production [9]. In this governorate, olive cultivation has as a consequence, considerable economic impact and displays social, environmental and landscape significance.

Unfortunately, these olive orchards are continuously damaged by major olive insect pests, namely the olive fly, *Bactrocera oleae* Gmelin (Diptera: Tephritidae), the olive moth, *Prays oleae* Bernard (Lepidoptera: Yponomeutidae), and the olive psyllid, *Euphyllura olivina* Costa (Homoptera: Psyllidae), which may cause high economic losses [10–13]; and plant pathogens such as *Fusicladium oleaginum* (Castagne) Ritschel & U. Braun (Dothideomycetes)*, Pseudocercospora cladosporioides* (Sacc.) U. Braun (syn. *Cercospora cladosporioïdes* (Sacc.) (Dothideomycetes), or *Verticillium dahliae* Kleb (Sordariomycetes) [14, 15]*.* The control of these pests and pathogens has been based on the application of chemical pesticides - e.g. those based on synthetic pyrethroids - characterized by their damaging effects to the ecosystems [16] as, e.g., pest-resistance problems [17], side-effects on auxiliary fauna [18, 19] and human health [20]. However, these practices are being replaced by biological control methods. Among the possible biological control methods, the use of entomopathogenic and the antagonistic fungi is considered as a promising strategy. The entomopathogenic activity of fungi as well as their antifungal activity depend on several factors including hydrolytic enzymes as amylases, proteases, lipases, chitinases, esterases, glucanases, and catalases [21–23]. Insects and fungi are embroiled in complex interactions between them. Enzymes such as amylases, proteases and lipases are implicated in the infective process of the insect by entomopathogenic fungi. In fact, lipases are involved in the adhesion of fungal spores on the insect cuticle, which is a mandatory pre-step that initiates the degradation of fatty acids and alkenes in the insect epicuticle [21]. Proteases intervene in the degradation of the proteinaceous material of insect and are considered as the most important enzymes for the infective process [22]. The amylase activity degrading glycogen of the insect tissues allows its use for the growth and invasion of entomopathogenic fungi [24].

In the present study, the mycobiota associated with olive trees and pests were investigated as part of a research effort targeting ecosystem-friendly approaches for olive tree pest and pathogen management. Fungal isolates from olive orchards were characterized by molecular methods, and investigated for their enzymatic, antifungal, entomopathogenic and phytopathogenic activities.

## Results

### Systematic characterization of the fungal isolates

Fungi isolation from the insect pest cadavers and soil samples in Taous and Torba allowed the identification of 34 fungal strains (Table 1, Fig. S1). Twenty fungal species were identified belonging to 6 different genera (*Alternaria*, *Aspergillus*, *Cladosporium*, *Fusarium*, *Lecanicillium*, *Penicillium*) and 5 families (Trichocomaceae, Pleosporaceae, Nectriaceae, Davidiellaceae, Cordycipitiaceae) by the comparison of their ITS1–5.8S-ITS2 sequences. All the 34 nucleotide sequences obtained from this region represented high degrees of identity with fungal ITS sequences available in the GenBank Database (scores > 98%).

**Table 1:** Fungal isolated from different olive orchards identified by sequence comparison with the BLASTn (NCBI GenBank database)

Family, *Genera* and *Species*	Sample isolation	mode of driving	Close relative research result (GenBank accession number)	Identity match (%)	No. of bp analyzed	Query cover	E-value
**Trichocomaceae**							
*Penicillium*							
*Penicillium chrysogenum*							
F3	Insect	Biological	*Penicillium chrysogenum* (KY445804.1)	100	300	100%	1e-80
F49’	Insect	Biological	*Penicillium chrysogenum* (KC811007.1)	98	100	100%	6e-49
*Penicillium crustosum*							
F14	Insect	Biological	*Penicillium crustosum* (MF072639.1)	99	170	98%	2e-69
F33	Insect	Conventional	*Penicillium crustosum* (MG596635.1)	100	130	100%	2e-51
*Penicillium freii*							
F29	Insect	Conventional	*Penicillium freii* (KY859388.1)	100	120	100%	2e-51
*Penicillium pinophilum*							
F36	Soil	Biological	*Penicillium pinophilum* (MF806019.1)	100	330	100%	5e-162
F38	Insect	Biological	*Penicillium pinophilum* (MF686817.1)	100	120	100%	2e-51
*Penicillium polonicum*							
F34	Insect	Biological	*Penicillium polonicum* (KX944174.1)	100	110	89%	1e-65
F41	Insect	Biological	*Penicillium polonicum* (KX944174.1)	100	120	100%	2e-51
*Penicillium verruculosum*							
F30	Insect	Conventional	*Penicillium verruculosum* (KY921956.1)	100	130	100%	2e-51
*Aspergillus*							
*Aspergillus calidoustus*							
F1’	Soil	Biological	*Aspergillus calidoustus* (KX610170.1)	100	150	100%	2e-74
F25	Soil	Biological	*Aspergillus calidoustus* (KX610170.1)	99	100	100%	2e-51
*Aspergillus nidulans*							
F23	Soil	Biological	*Aspergillus nidulans* (MG734752.1)	97	120	87%	6e-54
*Aspergillus niger*							
F46	Insect	Conventional	*Aspergillus niger* (MG759551.1)	100	120	100%	3e-50
*Aspergillus ochraceus*							
F21	Insect	Conventional	*Aspergillus ochraceus* (KX463003.1)	100	170	98%	7e-79
F57	Insect	Conventional	*Aspergillus ochraceus* (LT596572.1)	100	125	100%	2e-51
*Aspergillus pseudodeflectus*							
F13	Soil	Biological	*Aspergillus pseudodeflectus* (LN482409.1)	100	100	100%	8e-29
*Aspergillus tamarii*							
F60	Insect	Conventional	*Aspergillus tamarii* (MG682505.1)	99	230	98%	2e-120
*Aspergillus terreus*							
F58	Insect	Conventional	*Aspergillus terreus* (KX090315.1)	100	150	100%	9e-78
*Aspergillus ustus*							
F22	Soil	Biological	*Aspergillus ustus* (KF860885.1)	98	100	70%	8e-21
F26	Soil	Biological	*Aspergillus ustus* (KF860885.1)	99	120	94%	6e-27
F27	Soil	Biological	*Aspergillus ustus* (KF860885.1)	99	120	90%	2e-31
**Davidiellaceae**							
*Cladosporium*							
*Cladosporium cladosporioides*							
F18	Insect	Conventional	*Cladosporium cladosporioides* (KX255861.1)	99	150	100%	6e-71
F40	Insect	Conventional	*Cladosporium cladosporioides* (JX982429.1)	100	120	100%	2e-51
F50’	Insect	Biological	*Cladosporium cladosporioides* (KT898681.1)	100	115	100%	4e-48
F52’	Insect	Conventional	*Cladosporium cladosporioides* (KY381824.1)	99	335	100%	2e-149
*Cladosporium halotolerans*							
F45	Insect	Biological	*Cladosporium halotolerans* (KY445825.1)	99	120	100%	8e-50
*Cladosporium sphaerospermum*							
F17	Insect	Biological	*Cladosporium sphaerospermum* (KY046240.1)	99	250	100%	5e-105
F20’	Insect	Conventional	*Cladosporium sphaerospermum* (KY460929.1)	98	200	100%	3e-100
**Nectriaceae**							
*Fusarium*							
*Fusarium solani*-like							
F16	Insect	Conventional	*Fusarium solani* (KX235324.1)	99	200	99%	1e-55
F59	Insect	Conventional	*Fusarium solani* (MG757634.1)	100	100	100%	2e-51
**Pleosporaceae**							
*Alternaria*							
*Alternaria consortialis*							
F10	Insect	Conventional	*Alternaria consortialis* (KY458489.1)	99	160	100%	2e-51
F50	Insect	Conventional	*Alternaria consortialis* (KY458489.1)	100	140	100%	2e-51
**Cordycipitaceae**							
*Lecanicillium*							
*Lecanicillium aphanocladii*							
F28’	Insect	Conventional	*Lecanicillium aphanocladii* (KP689216.1)	99	250	100%	1e-132

The most strongly represented fungal taxonomic family was the Trichocomaceae. This family accounted for 22/34 (i.e. 64.7%) of the identified fungal strains that were assigned to 14 species. *Aspergillus* (*N* = 8 species), *Penicillium* (*N* = 6 species) and *Cladosporium* (*N* = 3 species) were the most prevalent genera, whereas *Cladosporium cladosporioides* (*N* = 4 isolates) and *Aspergillus ustus* (N = 3 isolates) were most prevalent at the species level.

The experimental plot of Taous that was managed conventionally and under artificial irrigation, displayed greater fungal richness than the Torba plot that was managed biologically and under rain-fed conditions. In fact, in the Taous plot, among a total of 17 fungal isolates obtained; 12 varied species were identified, belonging to 6 genera and 5 families. The family Trichocomaceae representing 47.0% (8/17) and the genus *Aspergillus* representing 29.4% (5/17) of the identified fungi were prevalent in this plot. Three genera were typically found only in the Taous plot, namely *Alternaria*, *Fusarium* and *Lecanicillium*.

Olive fly and olive psyllid pest cadavers displayed the greatest species richness, with 16 distinct species belonging to 6 genera and 5 families. Trichocomaceae was the most frequently detected family accounting for 53.8% (14/24) of the total of insect-derived fungal isolates, whereas *Penicillium* (9/24; 37.5%) and *Aspergillus* (5/24; 20.8%) were the most prevalent genera (Fig. S2).

The majority of fungal species (90%) tended to appear in very low numbers (singletons and doubletons) while only two taxa (10%) were represented by three or more isolates. The fungal diversity was evaluated through Simpson’s Index and values of this index showed that the two sites (Torba and Taous) have the same fungal diversity (0.95). The fungal biodiversity analysis with respect to the comparison between species of insects and soil, demonstrated that insects (0.96) had greater fungal diversity than soil (0.86).

### Screening for specific enzymes production

**Fig. 1:**
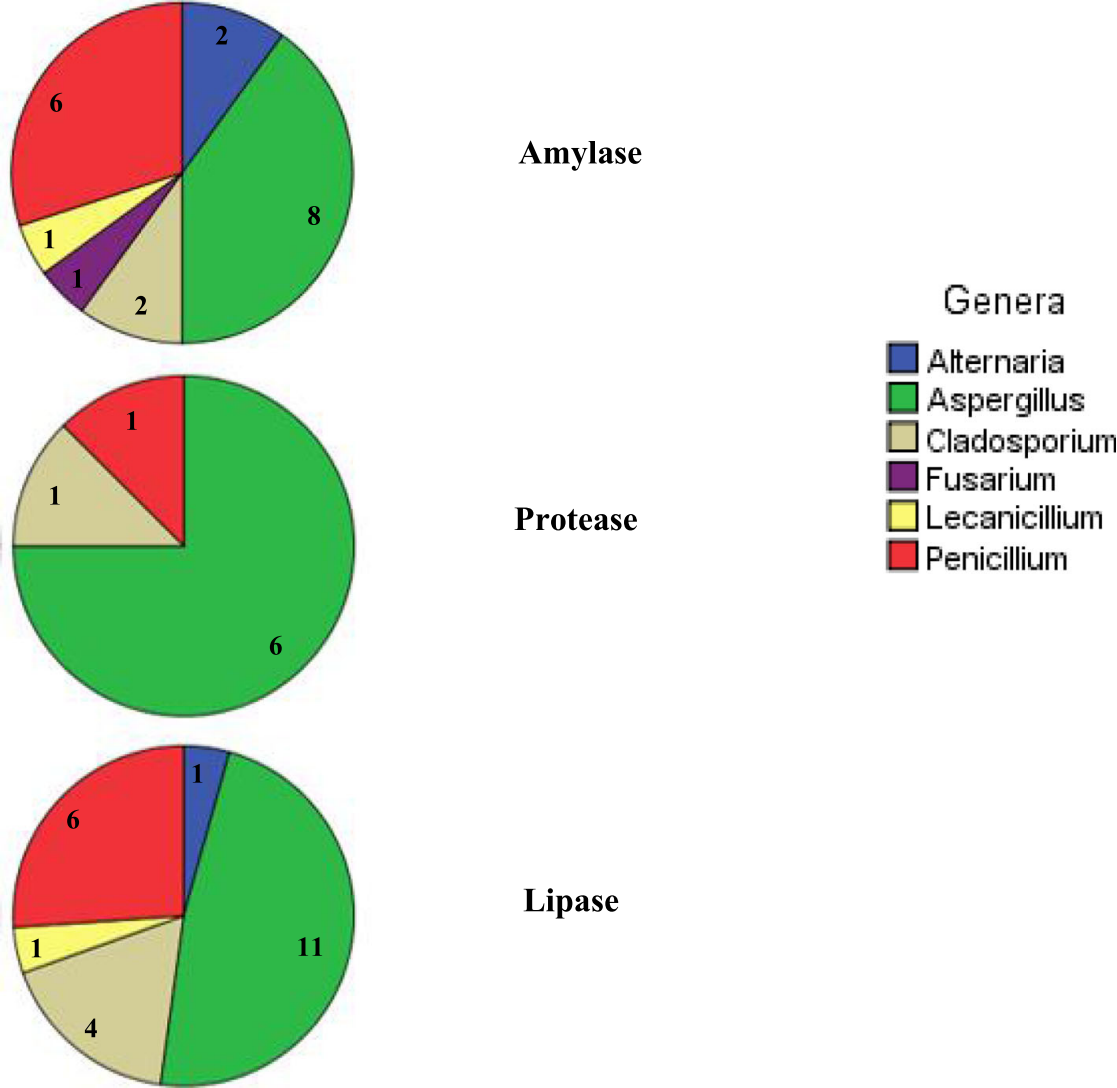
Genera enzymatic activity distribution of the fungal isolates related to Tunisian olive orchards. For each genus, the number of isolates producing extracellular enzyme was attributed

The 34 collected isolates were evaluated for their enzymatic activities, namely the amylase, protease and lipase enzymes (Fig. 1). Among these, amylase activities were most widespread being present in 58.8% (20/34) of the isolates. Most of amylase producing fungi belonged to the genera *Aspergillus* and *Penicillium*. Strains isolated from soil produced significantly more lipase than other isolates as, e.g., derived from olive fly and olive psyllid cadavers (*p* = 0.034).

Proteolytic activity was present in 23.5% (8/34) of the isolated fungi which showed their ability to produce extracellular protease by the degradation halos of casein on solid medium. *Aspergillus* was the genus able to degrade casein in all samples, with (statistically not significant) differences in the protease activity between isolates.

A high percentage of fungal isolates 67.6% (23/34) displayed an ability to produce extracellular lipase as determined by fluorescent halos around their colonies after irradiation with UV light. Among these lipase-producing isolates, 47.8% (11/23) were identified as *Aspergillus*, 26.1% (6/23) as *Penicillium* and 17.4% (4/23) as *Cladosporium*. *Aspergillus* was significantly the most abundant genus which comprised 75% (6/8) of the protease- and 47.8% (11/23) of the lipase-producing isolates with respectively significant differences of (*p* = 0.01) and (*p* = 0.03), respectively, to the representation of other genera.

### Antagonistic activity in vitro

The 34 fungi of our collection were evaluated for their antifungal activity using dual-culture tests against six reference strains representing the five fungal species *Aspergillus calidoustus*, *Penicillium chrysogenum* (two strains), *Alternaria consortialis*, *Aspergillus pseudodeflectus* and *Aspergillus tamarii* (Table 2). Almost two thirds of the olive tree or olive pest derived fungal isolates (22/34, 64.7%) belonging to 6 different genera and 16 species inhibited mycelial growth of at least one reference strain as compared with the untreated control. Isolates *Penicillium chrysogenum* F49’, *Aspergillus ochraceus* F57 and *Fusarium solani-*like F59 showed the highest antifungal activity (4/6, 66.7%) in terms of the number of inhibited reference strains, and *Penicillium chrysogenum* F49’ presented the strongest antifungal activity by producing the largest inhibition zones against 4 target fungi. Among the 34 isolates, 23.5% (8/34), 35.3% (12/34), 41.2% (14/34), 5.9% (2/34), 8.8% (3/34), and 23.5% (8/34) displayed antagonistic activity against *A. calidoustus*, *P. chrysogenum*1, *A. consortialis*, *A. pseudodeflectus*, *P. chrysogenum*2 and *A. tamarii,* respectively. The antibiosis was significantly observed as mode of action employed by both *Fusarium* and *Lecanicillium* fungal strains assayed against the *A. consortialis* reference strain *(p* = 0.046). The dual culture of *P. chrysogenum* F49’ and *A. consortialis* recorded the highest inhibition zone.

**Table 2:** Antagonistic activity of our collected fungal isolates against 6 pathogenic fungal isolates

Antagonistic Fungal isolates	Fungal pathogens					
	*Aspergillus calidoustus*	*Penicillium chrysogenum*1	*Alternaria consortialis*	*Aspergillus pseudodeflectus*	*Penicillium chrysogenum*2	*Aspergillus tamarii*
*Penicillium chrysogenum*						
F3	+	–	++	–	–	–
F49’	+	++	+++	+++	–	–
*Penicillium crustosum*						
F14	+++	+	+++	–	–	–
F33	–	–	–	–	–	–
*Penicillium freii*						
F29	–	–	–	–	–	–
*Penicillium pinophilum*						
F36	–	++	++	–	–	+++
F38	–	–	–	–	–	–
*Penicillium polonicum*						
F34	++	–	+++	–	–	–
F41	+	–	–	–	–	++
*Penicillium verruculosum*						
F30	–	–	–	–	–	–
*Aspergillus calidoustus*						
F1’	–	++	–	–	–	–
F25	–	+++	–	–	–	–
*Aspergillus nidulans*						
F23	–	–	++	–	–	–
*Aspergillus niger*						
F46	+	+	++	–	–	–
*Aspergillus ochraceus*						
F21	–	+	–	–	–	++
F57	–	+	+	–	+	+
*Aspergillus pseudodeflectus*						
F13	++	+++	+	–	–	–
*Aspergillus tamarii*						
F60	–	–	++	++	+	–
*Aspergillus terreus*						
F58	–	–	+	–	–	–
*Aspergillus ustus*						
F22	–	–	–	–	–	–
F26	–	+++	–	–	–	++
F27	–	+++	–	–	–	++
*Cladosporium cladosporioides*						
F18	–	–	–	–	–	–
F40	–	–	–	–	–	–
F50’	–	–	–	–	–	–
F52’	–	–	–	–	–	–
*Cladosporium halotolerans*						
F45	–	–	–	–	–	–
*Cladosporium sphaerospermum*						
F17	–	–	–	–	–	–
F20’	–	–	–	–	–	–
*Fusarium solani-*like						
F16	–	–	++	–	–	–
F59	–	++	++	–	+	++
*Alternaria consortialis*						
F10	+++	–	–	–	–	–
F50	–	–	–	–	–	+
*Lecanicilliumaphanocladii*						
F28’	–	–	+	–	–	–

The activity was detected for clear halo zones around the colonies. The inhibition zone diameters varied from 0 to 40 mm. The inhibition zones diameterincrease concurrently. + [5–10], ++ [11–20], +++ [21–40], − no zones of inhibition

### Entomopathogenic activity

**Fig. 2:**
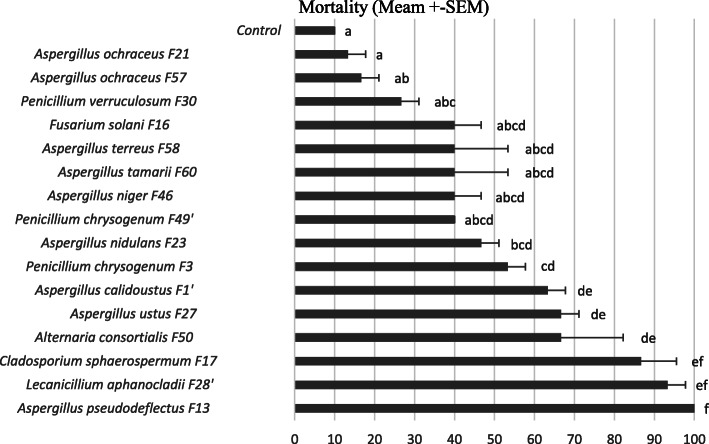
Mean mortality rate of forth instar *Ephestia kuehniella* larvae fed with semolina mixed with conidial suspension of fungi at concentration adjusted to 10^7^ conidia/ml, 7 days after treatment. Bars followed by letters are significantly different (*p* < 0.05), according to Tukey test

The 16 tested fungal isolates displayed significant differences in their entomopathogenic activity as determined 7 days after treatment by the number of dead fourth instar larvae fed on a diet containing a fungal suspension (10^7^ Conidia/ml), with *p* < 0.05 (Fig. 2). One-way ANOVA was done to test significant differences between the means of mortality percentage. Results studying the effect of the treatment are significant (*p* < 0.05) as compared to the negative control. The mortality percentage of L4 larvae fed semolina contaminated with the fungal suspension varied from 13% up to 100%. From our fungal isolates, *Aspergillus pseudodeflectus* F13 and *Lecanicillium aphanocladi* F28’ were the most pathogenic with mortality rates of 100 and 93.3%, respectively, followed by *Cladosporium sphaerospermum* F17 (86.6%). Treated cadavers incubated in adequate conditions resulted in confirming the fungal infection causing the death of the insect.

### Phytopathogenic activity

The study of the phytopathogenic activity of 34 distinct fungal isolates, using the excised shoot assays, was evaluated in terms of browning on cortex and vascular tissues 28 days after inoculation. Whereas, 11.8% (4/34) of the tested isolates, namely *Penicillium crustosum* F14, *Penicillium polonicum* F34, *Aspergillus ochraceus* F57 and *Aspergillus terreus* F58, have produced cortex browning compared to control shoots, 14.7% (5/34), more exactly *Alternaria consortialis* F10, *Aspergillus ochraceus* F21, *Aspergillus ustus* F22, *Aspergillus nidulans* F23 and *Alternaria consortialis* F50, were found to cause vascular tissue browning (Table 3).

**Table 3:** Phytopathogenic activity, of fungal isolates showing cortex browning or vascular tissue browning, using excised shoot assays 28 days after inoculation based on tissues browning

Fungal isolates displaying phytopathogenic activity	Phytopathogenic activity	
	Cortex Browning	Vascular Tissues Browning
*Penicillium crustosum* F14	+	–
*Penicillium polonicum* F34	+	–
*Aspergillus nidulans* F23	–	+
*Aspergillus ochraceus* F21	–	+
*Aspergillus ochraceus* F57	+	–
*Aspergillus terreus* F58	+	–
*Aspergillus ustus* F22	–	+
*Alternaria consortialis* F10	–	+
*Alternaria consortialis* F50	–	+

## Discussion

### Richness of fungal taxa

In this work, the diversity of fungi isolated from major olive pests and soil samples was evaluated. The molecular identification using the ITS marker presents 20 fungal species, belonging to 6 genera and 5 families. To support the identification of *Fusarium* species, more genetic markers should be included. In fact, ITS did not present a good molecular marker to identify species of *Fusarium* since this region is rather well-conserved in the genus [25].

The genera *Aspergillus* and *Penicillium* were most abundant in the collected samples comprising 64.7% (22/34) at the strain and 70% (14/20) at the species level independently on production guidelines. These observations are in line with studies emphasizing the largest widespread of *Penicillium* and *Aspergillus* identified as the dominant genera in most of the ecosystems and were the most ubiquitous fungal species in nature (soils, plants and agricultural communities) [13, 26–28].

The identification of this number of fungal taxa describing enzymatic, antagonistic, phytopathogenic and entomopathogenic features was variable. These findings are in line with previous studies showing that the fungal diversity was maintained under organic and integrated production guidelines. In fact, in the literature it was reported that the expected fungal diversity performed on a conventional orchard would have been lower than organic and integrated orchards which creates a healthier and safer environment with higher biological diversity [13, 29, 30]. Nevertheless, in our study the fungal abundance and richness were probably positively affected by the presence of water and humidity due to irrigation in the conventional orchard. These data are consistent with studies that suggest dew duration and relative humidity are critical parameters for predicting the fungal biomass of the soil [31, 32].

Insect cadavers displayed greater species diversity than soil samples, with 16 different species versus 5 species, respectively. Among the 16 insect-derived species, 15 (93.7%) were found only in olive fly or olive psyllid cadavers, namely *A. consortialis*, *A. niger*, *A. ochraceus*, *A. tamarii*, *A. terreus*, *C. cladosporioides*, *C. halotolerans*, *C. sphaerospermum*, *F. solani-*like, *L. aphanocladii*, *P. chrysogenum*, *P. crustosum*, *P. freii*, *P. polonicum* and *P. verruculosum,* whereas, the *Aspergillus species A. calidoustus*, *A. nidulans*, *A. pseudodeflectus,* and *A. ustus* were found only in soil.

### Biotechnological properties of fungal isolates

#### Enzymatic activities

Not all isolates of the same fungal species necessarily produce the same enzyme and have the same intensity of the enzymatic activities. These properties are strain dependent [33]. In fact, the enzymatic production differs among fungi and is related to their natural habitat or ecological niche [34].

Our results showed that amylolytic activity was the most common among all our collected strains. These results are consistent with previous studies showing that amylase was the predominant secreted enzyme of endophytic fungi isolated from soybean [35]. Moreover, *Penicillium* species have been reported to be prominent producers of amylolytic activities [36], as is the case for *Aspergillus* fungi isolated from different seeds, soils or from *Morinda citrifolia* [37–40]. Consistently, among the fungal isolates investigated in this study, those belonging to the genera *Aspergillus* and *Penicillium* were the most prominent amylase producers.

In addition to the amylolytic activity, *Aspergillus* was significantly the most abundant genus producing protease and lipase activities as compared to the other genera identified here. Consistently, previous studies with 110 fungal isolates of *Aspergillus*, *Fusarium*, *Cladosporium* and other genera had revealed that 73% of them produced protease, lipase and urease [41]. Predominant among the lipase and protease producing isolates were *Aspergillus* sp., *Cladosporium* sp., *Myrothecium* sp. and *Fusarium* sp..

In addition to their well-established nutritional function [36], amylase, protease and lipase activities could play an important role in fungal ecology and during microbial infection exhibiting antagonistic, entomopathogenic, and phytopathogenic features. Secreted enzymes such as amylases, proteases and lipases are known to be implicated in the interaction between insect hosts and entomopathogenic fungi. Lipases are involved in the adhesion of fungal spores on the insect cuticle, amylase allows degradation of glycogen from insect tissues for growth and invasion, and proteolytic degradation has been identified as a key factor in fungal pathogenesis, including several species of *Aspergillus* [36] and the infective degradation of proteinaceous material of the host cuticle by infection hyphae of entomopathogenic fungi [21, 22].

#### Antagonistic activity

Increasing antifungal activity is a potentially useful and effective strategy for fungal biocontrol. A fungal biocontrol agent may act against fungal pathogens by using antibiosis which is defined as the capacity of producing different antibiotics probably involved in the suppression of competitors. This mode of action is exercised by a broad number of fungal bio-controllers [42]. Moreover, other studies reported that the antagonistic activity of fungi were associated to competition for nutrients and space [43–45].

In this study, fungal isolates have clearly shown their antagonistic properties against the six tested fungal reference strains. Antagonistic activity of *Penicillium* species against these fungi was characterized by the production of toxic metabolites. According to previous reports, filamentous fungi, including some of the strains tested in our study as *Penicillium chrysogenum* F49’, *Aspergillus ochraceus* F57 and *Fusarium solani-*like F59 may restrict and stop fungal growth. These filamentous fungi such as *Aspergillus* and *Penicillium* are known as antimicrobial due to their ability to produce toxic secondary metabolites including hydrolytic enzymes such as protease, amylase and lipase [46].

The susceptibility of *Alternaria consortialis* to 14 of the fungal isolates as, e.g., *Penicillium chrysogenum*, *Penicillium crustosum*, *Penicillium pinophilum*, and *Penicillium polonicum* tested in this study is consistent with previous studies reporting the antagonistic activity of antifungal compounds produced by *Penicillium* sp., (Macropherin A, Atrpinin A, Botryodiplodin and Brefeldin A) which allowed the inhibition of *Alternaria* sp. [3, 47].

#### Entomopathogenic activity

From the fungal taxa identified in this study, *Aspergillus pseudodeflectus* F13 and *Lecanicillium aphanocladii* F28’ were found highly pathogenic to *Ephestia kuehniella* larvae. Previous studies have associated *Aspergillus pseudodeflectus* with entomopathogenic activity and the stimulation of fungal aflatoxin production by the insect host. It has been shown that the addition of aflatoxin to the food of insect larvae kills them and promotes the growth of the fungus [48, 49]. Consequently, experiments to evaluate its infecting ability towards vertebrates have to be performed.

According to our knowledge, *Lecanicillium aphanocladii* fungi have not previously been recorded in association with olive pests. Isolate *L. aphanocladii* F28’ was characterized as a potent entomopathogen causing a high percentage (75%) of larvalmortality. *L. aphanocladii* has previously been found associated with diseased and dead lepidopteran insects [50], and there appears to be clear link between the isolation from insect cadavers and entomopathogenic activity. The insect surface sterilization before fungal isolation as well as insect susceptibility bioassays confirmed this activity. The efficiency of *Lecanicillium aphanocladii* to parasitize dipteran insects as, e.g., mosquito larvae has also been reported [51, 52]. Furthermore, *Lecanicillium* spp. was used for 15 commercial preparations.

#### Phytopathogenic activity

In addition to these potential biocontrol agents which have a role in limiting pests, other fungi have antagonistic features for limiting the occurrence of phytopathogenic fungi in olive groves. Among the phytopathogenic fungi we have identified *Alternaria consortialis* F50 which have showed vascular tissue browning. This is consistent with the previous identification of *Alternaria* species as the cause of olive spoilage and a disease on olive shoots grown under greenhouse conditions [13]. Moreover, isolate *Alternaria consortialis* F50 has been shown to display entomopathogenic activity against *E. kuehniella* (larval mortality of 66.6%). These results are corroborated by the findings of previous studies reporting the efficiency of *Alternaria* spp. as entomopathogenic fungi where the insect may ingest them through infected leaves [53].

*Alternaria* strains displaying entomopathogenic, but lacking phytopathogenic activity could be used in pest management as whole organisms [54]. However, with both entomopathogenic and phytopathogenic activities being recorded as is the case for strain *A. consortialis* F50, investigation and identification of the insecticidal principle or mode of action could lead to the development of derived pest control agents not requiring application of the living fungus [55].

## Conclusions

The present study describes for the first time the fungal diversity obtained from dead insect pests collected from Tunisian olive groves. The screening of secreted fungal enzymes showed highly variable activity profiles which might have impact for improving technological processes. With respect to the possible development of biological control agents, 50% of the fungal species from our collection showed entomopathogenic or antagonistic activity and were not phytopathogenic as determined in terms of browning length on cortex and vascular tissues. From the fungal taxa identified in this study, *Aspergillus pseudodeflectus* F13 and *Lecanicillium aphanocladii* F28’ appear the most noticeable as they combine high entomopathogenic potential with lack of phytopathogenic activity against olive trees and – in the case of *A. pseudodeflectus* - antagonistic activity against phytopathogenic fungi. As endemic isolates well adapted to the regional climatic conditions, these strains could be promising in view of the development of protection of olive orchards after their insecticidal bioassays on the olive major pests.

## Methods

### Field sites and fungal isolation

Samples were randomly collected from 2 different olive groves located in two distinct geographical zones (Taous and Torba) of Sfax governorate, Tunisia (GPS coordinates: (34°55′17″N, 10°36′47″W; 34°50′56.9″N10°27′33.6″E). These samples were collected once per month in the period ranging from December 2016 to February 2017. At each site, around twenty olive fruits and twenty branches infested respectively by the olive fly, *B. oleae,* and the olive psyllid, *E. olivine,* as well as 100 g of soil (from 10 cm of the upper soil layer) were sampled from two sampling locations within the olive orchards. Each sample was packaged in a sterile plastic bag (31 × 20 cm), transported to the laboratory under aseptic conditions and stored at 4 °C until the isolation procedure of fungi was carried out. The olive orchards were chosen according to the pest and pathogen management strategy practiced: Whereas the experimental plot at Taous was maintained under conventional control measures and in irrigated, the Torba plot was conducted according to the organic farming system under rain-fed conditions.

Sampled insect cadavers were surface sterilized in a 70% ethanol solution for 1 min in laminar chamber. The surface-sterilized insects were rinsed 3 times with sterile water for 1 min and blotted dried on sterile paper towels. Each sample was ground using a micro-pestle in sterilized distilled water. The soil samples were treated as follows: 10 g of soil was dissolved in 90 ml of sterile distilled water and mixed with a stomacher. Supernatants were diluted 10-fold and 100 μl of each dilution was spread on the potato dextrose agar media (PDA: 4 g l^− 1^ Potato extract, 20 g l^− 1^ Dextrose, 16 g l^− 1^ Agar). A total of 34 pure fungal cultures were isolated after continuous sub-cultivation. The pure cultures were recorded and maintained on PDA in active form for further investigation. For long-term storage, the fungal colonies were stored in paraffin oil at − 20 °C.

### Identification of fungal isolates

The isolates were initially identified based on morphological structures (color, texture of the mycelia and spore formation) performed under microscopic observation of fresh cultures [56]. For fungal DNA extraction, the mycelial mass obtained from the culture plate was placed in a 2 ml tube, submerged in liquid nitrogen and then ground using a micro-pestle in lysis buffer. The extraction protocol is continued using the DNeasy Plant Mini kit (Qiagen® GmbH, Hilden, Germany) following manufacturer’s instructions. The quality of the genomic DNA was evaluated by a NanoDrop NT-100 UV spectrophotometer (NanoDrop technologies®, Montchanins, USA).

Molecular identification of isolates was made by PCR amplification of the internal transcribed spacer (ITS) region of the nuclear rRNA operon comprising both the ITS1 and ITS2 elements separated by the 5.8S rRNA gene [57] using the fungal specific primers ITS5 (5′-GGAAGTAAAAGTCGTAACAAGG-3′) and ITS4 (5′-TCCTCCGCTTATTGATATGC-3′) [58]. The amplification was performed in 50 μl reaction volume mixture containing 42.25 μl of H_2_O, 5 μl of Taq PCR Buffer (10X), 1 μl of dNTPs mix (200 μM), 0.25 μl of each primer (0.4 μM), 0.25 μl of Taq Polymerase (1.25 units/ 50 μl PCR) (New England Biolabs®, Germany) and 1 μl DNA template. The PCR was performed as follows: initial denaturing step at 95 °C for 3 min, followed by 35 cycles of 95 °C for 45 s, 52 °C for 45 s, and 68 °C for 2 min, and a final extension step at 68 °C for 5 min. PCR products were isolated by electrophoresis on a 1% agarose gel and purified using PCR Purification Kit (Qiagen® GmbH, Hilden, Germany) following the manufacturer’s instructions.

### Phylogenetic analysis

The amplified ITS products were sequenced using ITS4 and ITS5 primers. Sequencing was performed by Starseq® GmbH (Mainz, Germany). The ITS sequence information was used to match the most closely related fungal isolates with the NCBI BLAST algorithm from the GenBank database (http://www.ncbi.nlm.nih.gov). The analysis of the ITS sequences were performed using the MEGA 6 program [59] (http://www.megasoftware.net).

### Screening for extracellular enzyme production

Qualitative tests were used to evaluate the production of fungal extracellular enzymes of interest in industrial biotechnology. The screened enzymes were lipase, protease and amylase.

Lipase production was tested on PDA (1 l) supplemented with olive oil (16.0 ml), and Rhodamin solution (10 mg.ml^− 1^) (Sigma Aldrich®, Germany). After development of the isolates on agar at optimal conditions, lipase activity was detected by irradiating with Ultra Violet light at 350 nm. Positive reaction is accompanied by the presence of precipitates around the fungal colony [60].

Protease production was revealed on skim milk agar. To determine the proteolytic potential, fungi were grown at 25 °C for 72 h. The enzyme production was assessed by the appearance of a clear zone corresponding to casein hydrolysis surrounding the fungal colony [61].

To check for amylase activity, fungi were incubated in PDA medium supplemented with starch (1%). After incubation and fungal growth an iodine solution (0.3 g iodine and 0.6 g.l.^− 1^) was added to the plate. Amylase production is shown by the presence of a clear halo due to the degradation of starch and failure to form the colored starch-iodine intercalation complex [62].

### Detection of antagonistic activity in vitro

The in vitro antagonistic activity of the collected fungal isolates was tested against the following pathogenic fungi provided by the Olive Tree Institute: *Aspergillus calidoustus, Aspergillus pseudodeflectus, Aspergillus tamarii, Alternaria consortialis* and *Penicillium chrysogenum.* These latter represent known pathogens of the olive tree and are, therefore, main target organisms for biocontrol in olive orchards.

The antagonism assays were conducted using dual-culture tests. Petri dishes containing PDA medium were first inoculated with the conidia of an actively growing fresh fungal culture. The fungal suspension was standardized to approximately 10^6^ conidia/ml using Malassez lame under microscope (Leitz Dialux 9 40 EB). They were obtained by scraping conidia from 15-day-old cultures of fungal isolates on PDA into an aqueous solution of 2% Tween 80 then filtered through cheesecloth to remove mycelium. 100 μl of each fungal suspension was spread onto the surface of the Petri dishes. Then, mycelial disc (10 mm in diameter) cut from the antagonistic fungal culture was positioned in the center of the plate and incubated at 25 °C in the dark for 7 days. The assay was concluded when the pathogen’s radial growth reached the antagonist. The only type of interaction monitored was the antibiosis determined by the presence of inhibition zones, which was measured and considered as indication for antifungal activity.

### Screening for Entomopathogenic activity

The pathogenicity of fungal isolates was tested on the factitious host *Ephestia kuehniella* Zeller (Lepidoptera: Pyralidae)*.* This moth was reared on whole wheat semolina in the Laboratory of Improvement and Protection of Olive Tree Genetic Resources, Olive Tree Institute (Sfax, Tunisia). Fourth instar larvae were used for fungal susceptibility tests. For each assay, 10 larvae were fed with 1 g of semolina mixed with 500 μl of a culture suspension (1 × 10^7^ conidia ml^− 1^). The conidial suspension of each fungal strain was determined under the microscope using a Neubauer hemocytometer and adjusted to the cited concentration; a diet with 1 g sterile semolina mixed with 500 μl of sterile water was used as the control. Three replicates were performed for each fungal isolate, including the control [46]. The cumulative mortality was recorded by counting the number of dead larvae after 7 days of incubation under controlled conditions (temperature 22–25 °C, 70 ± 10% RH, natural light). To satisfy Koch’s postulates, the dead larvae were treated as follow: firstly, larval cadaver surfaces were sterilized with 5% sodium hypochlorite, rinsed twice and dried on filter paper, then incubated at 25 °C under saturating conditions to allow the development of the tested fungi on the surface of the insect cadaver. The conformity of our results was justified after the tested fungi grew, were transferred to fresh PDA medium and incubated at 25 °C. The obtained fungi were determined by microscopy (Leitz Dialux 40 EB).

Data were analyzed using ANOVA and values (mean ± standard error) were compared using Tukey test and differences were statistically significant at *p* < 0.05. Statistical analyses were performed using SPSS software for version 19.0 (SPSS Inc., Chicago).

### Evaluation of Phytopathogenic activity

Phytopathogenic activity of fungal isolates against olive trees was evaluated using the excised shoot assays [63]. Shoots of approximately 250 mm length and 15 mm diameter were collected from olive trees. Shoot surfaces were sterilized for 10 min with 10% sodium hypochlorite solution rinsed with sterile water and dried using sterile paper towels. A plug of the same size (5 × 5 mm) of 7 days-old colony was incubated in the middle of the shoot after removing the cortex flap (5 × 8 mm) by a sterile scalpel. Five shoots were inoculated with each isolate. Inoculated shoots were then kept at 28–32 °C, 70 ± 10% RH, natural light for 28 days. The assays were performed in 5 replicates. To reveal the trunk browning, the bark of the inoculated shoots was scraped with a sharp knife. Inoculated shoots were longitudinally cut at whole length into two halves in order to check wood browning in the internal vascular tissues. To comply with Koch’s postulates, sections of brown tissues were cultured on PDA and incubated at 25 °C for 7 days. Fungal cultures that grew up were identified microscopically (Leitz Dialux 40 EB).

## Supplementary information

**Additional file 1: Supplementary Figure 1.** Taous is an experimental orchard of the Olive Tree Institute, with an area of 126 ha. It contains olive, almond and pistachio fields. Olive cultivations include several varieties. In this study, insects were sampled from the variety Chemlali grown under artificial irrigation. The plantation density is 69 plants per hectare, with a spacing of 12x12m. Regular monitoring of the major olive pests is performed and organophosphate contact insecticides are applied if required. Torba is a private olive orchard with an area of 50 ha. Insects and soil were sampled from the olive cv. Chemlali cultivated under rain-fed conditions. The density of plantation is 17 plants per hectare, with spacing of 24x24m. Regular monitoring of the major olive pests is performed and mass trapping is conducted if required. The Images depicted in Figure are our own.

**Additional file 2: Supplementary Figure 2.** Sunburst chart showing the total relative abundance of fungal species and their corresponding family detected in two olives orchards in Tunisia.

## Data Availability

The datasets used and/or analysed during the current study are available from the corresponding author on reasonable request.

## Abbreviations

ITS: Internal Transcribed Spacer
DNA: Deoxyribonucleic acid
e.g.: Exempli gratia
UV: Ultraviolet
PDA: Potato Dextrose Agar
PCR: *Polymerase Chain Reaction*
rRNA: Ribosomal ribonucleic acid
