# Supplementary figures and images for: Identification of fungi in Tunisian olive orchards: characterization and biological control potential
Source: BMC Microbiol. 2020 Oct 12;20:307. doi: 10.1186/s12866-020-01997-z (PMC7552492; doi:10.1186/s12866-020-01997-z)

**Supplementary Figure 1:**

**
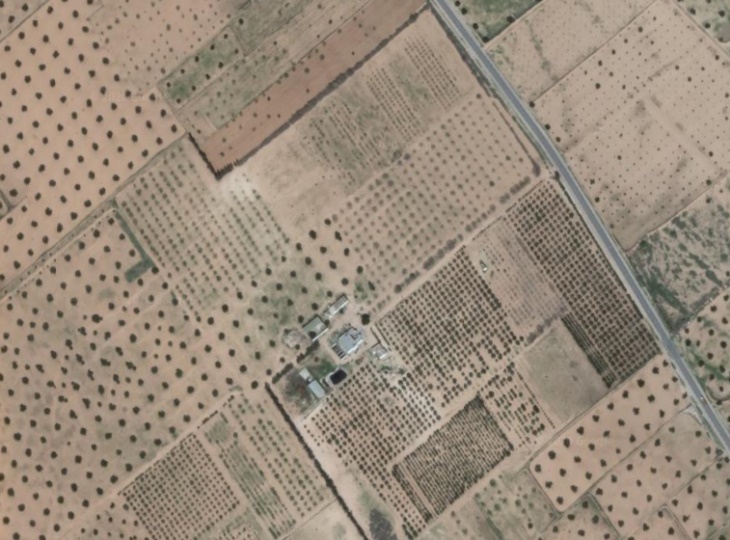

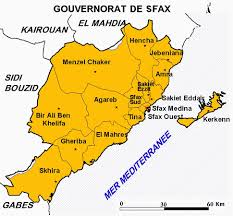

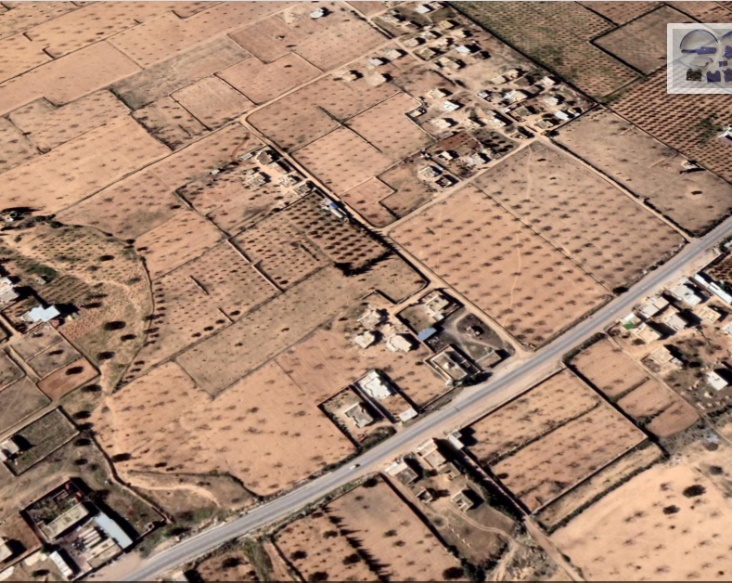
**

Supplement: Supplementary file 1 — Additional file 1: Supplementary Figure 1. Taous is an experimental orchard of the Olive Tree Institute, with an area of 126 ha. It contains olive, almond and pistachio fields. Olive cultivations include several varieties. In this study, insects were sampled from the variety Chemlali grown under artificial irrigation. The plantation density is 69 plants per hectare, with a spacing of 12x12m. Regular monitoring of the major olive pests is performed and organophosphate contact insecticides are applied if required. Torba is a private olive orchard with an area of 50 ha. Insects and soil were sampled from the olive cv. Chemlali cultivated under rain-fed conditions. The density of plantation is 17 plants per hectare, with spacing of 24x24m. Regular monitoring of the major olive pests is performed and mass trapping is conducted if required. The Images depicted in Figure are our own. [file 12866_2020_1997_MOESM1_ESM.docx]

**Supplementary Figure 2:**


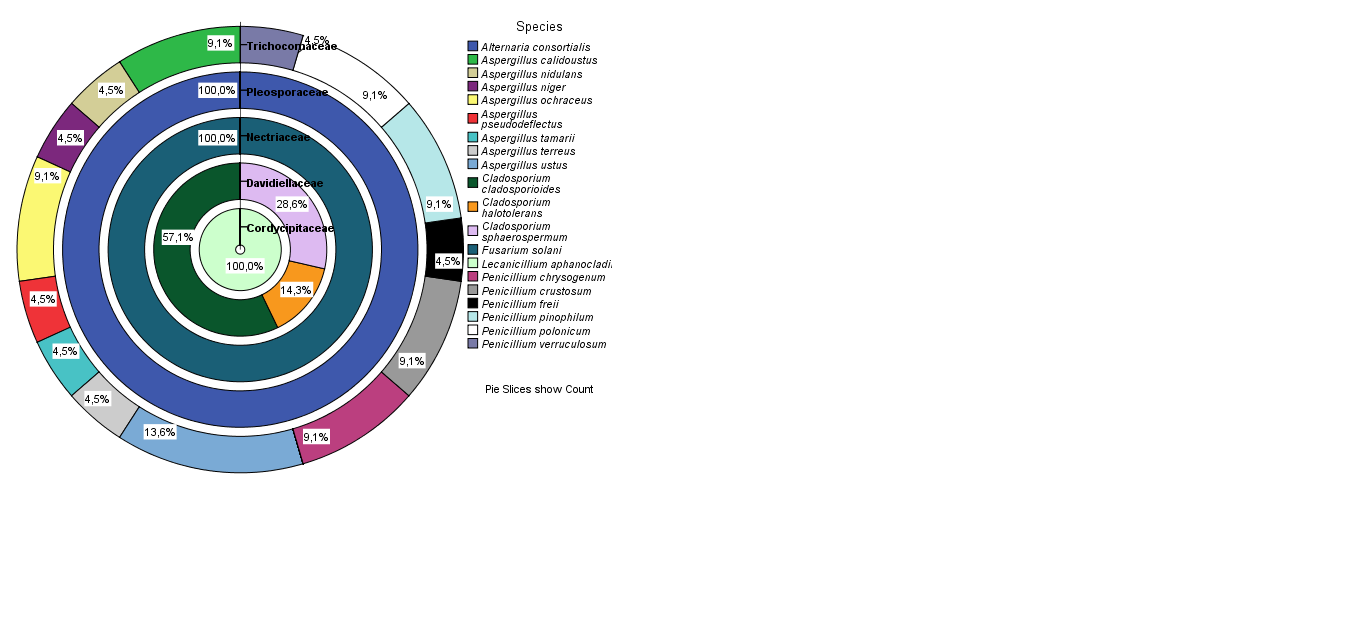

Supplement: Supplementary file 2 — Additional file 2: Supplementary Figure 2. Sunburst chart showing the total relative abundance of fungal species and their corresponding family detected in two olives orchards in Tunisia. [file 12866_2020_1997_MOESM2_ESM.docx]
